# Teleconsultation demand classification and service analysis

**DOI:** 10.1186/s12911-021-01610-x

**Published:** 2021-08-21

**Authors:** Wenjia Chen, Jinlin Li

**Affiliations:** School of Management and Economics, Beijing Institute of Technology, Beijing, 100081 China

**Keywords:** Teleconsultation, Demand classification, An ensemble hierarchical clustering method, Service efficiency and quality

## Abstract

**Background:**

To enhance teleconsultation management, demands can be classified into different patterns, and the service of each pattern demand can be improved.

**Methods:**

For the effective teleconsultation classification, a novel ensemble hierarchical clustering method is proposed in this study. In the proposed method, individual clustering results are first obtained by different hierarchical clustering methods, and then ensembled by one-hot encoding, the calculation and division of cosine similarity, and network graph representation. In the built network graph about the high cosine similarity, the connected demand series can be categorized into one pattern. For verification, 43 teleconsultation demand series are used as sample data, and the efficiency and quality of teleconsultation services are respectively analyzed before and after the demand classification.

**Results:**

The teleconsultation demands are classified into three categories, erratic, lumpy, and slow. Under the fixed strategies, the service analysis after demand classification reveals the deficiencies of teleconsultation services, but analysis before demand classification can’t.

**Conclusion:**

The proposed ensemble hierarchical clustering method can effectively category teleconsultation demands, and the effective demand categorization can enhance teleconsultation management.

**Supplementary Information:**

The online version contains supplementary material available at 10.1186/s12911-021-01610-x.

## Introduction

When facing different-pattern demands, an effective way to improve service management is demand categorization, which is widely used in spare parts management [1]. Learning from the successful experience of demand classification in spare parts management, teleconsultation management can be enhanced by demand categorization. The teleconsultation demands of medicine departments are different, but teleconsultation demand classification, to our best knowledge, hasn’t been reported in the existing literature. Even teleconsultation relevant operations and management research were few reported in the literature. These relevant researches included daily demand forecasting [2], resource allocation [3], service quality assessment [4], appointment and scheduling [5], workload management [6], and the government subsidy policy [7].

Guiding the demand forecasting and service scheduling of teleconsultation to improve the management, demand categorization is necessary for two reasons. On the one hand, demand patterns, like the intermittent, can influence the selection of forecasting methods [1, 8]. One method has different forecasting accuracy when applying to different pattern demand data. On the other hand, similar to the different inventory strategies for different-pattern demands in inventory management, there should be different scheduling strategies for teleconsultation services of different-pattern demands [9]. Therefore, this study aims at improving teleconsultation service by demand classification and service analysis before and after demand classification.

The main contributions of this paper are described as follows.

1. A novel ensemble hierarchical clustering method is proposed for teleconsultation demand classification. Four hierarchical clustering methods, one-hot encoding, cosine similarity, K-means clustering, and network graph are applied in the proposed method.
2. Actual teleconsultation demand data is used as sample data to prove the effectiveness of the proposed clustering method. By the proposed clustering method, teleconsultation demands are classified into five groups in three patterns.
3. The importance of teleconsultation demand classification is proved by the comparison of service efficiency and quality before and after the demand classification under the fixed service strategies. After demand classification, the service analysis reveals the service deficiencies of different-pattern demands, providing suggestions for service improvement.

The rest of the paper is organized as follows. “Literature review” section reviews related literature. “Methods” section introduces the proposed method. “Data description and experimental design” section describes the experimental data and designs. “Results and discussions” section presents the results and discussions. “Conclusions” section gives a conclusion.

## Literature review

Previous studies of demand classification focus on the area of spare parts in the stock-keeping unit (SKU) [10, 11]. For universally applicable classification, a nomenclature system was introduced in [12]. The framework contains two factors, the mean inter-demand interval and the coefficient of variation of demand size. The combinations of these two factors lead to four categories: erratic, lumpy, smooth, and slow [13]. Erratic categories have high demand size variability and low levels of intermittence. Lumpy categories have high demand size variability and high levels of intermittence. Smooth categories have low demand size variability and high levels of intermittence. Slow categories have low demand size variability and low levels of intermittence.

In the studies of demand classification of spare parts, specific cut-off values are used in the classification methods [1]. However, there is no consensus on a cut-off value for classification criteria, rather it depends on the type of time series, related industry, and demand volume. Teleconsultation demand is a daily time series in healthcare. Therefore, the existing demand classification method isn’t suitable for teleconsultation demand classification. Despite this, the conceptual framework for SKU classification and corresponding criteria can be used in teleconsultation demand classification [11]. The available criteria are summarized in Table 1.

**Table 1:** Overview of the main spare parts classification researches

References	Classification criteria	Cut-of values of criteria	Classification results	Assumption
[14]	The mean number of lead times between demands	0.5, 0.7, 2.8	Three categories: sporadic, slow-moving, smooth	Poisson demand arrivals
	The ‘lumpiness’ of the demand			
[15]	The ratio of average inter-order interval and forecast review periods	1.25	–	Poisson demand arrivals
[16]	Transaction variability	0.1, 0.53, 0.74	Five categories: smooth, irregular, slow-moving, erratic, highly erratic	–
	Demand size variability			
	Lead-time variability			
[13]	The square coefficient of variation of demand sizes, The average inter-demand interval	0.49, 1.32	Four categories: erratic, lumpy, smooth, slow	Bernoulli demand arrivals
[12]	The number of zeroes in the last 13 periods	5–8 for Croston method	Intermittent	–
		2–4 for the SBA method	Non-intermittent	
[17]	Frequency	Pareto classification (80%, 95%, 100%)	Three categories: A, B, C	–
	Demand value			
[18]	The item first occurred; The average quarterly demand; The average time between demands (quarter); Standard deviation to mean ratio	3, 8; 25; 2; 1.75, 2.50	Lumpy	Outliers must be removed
			Erratic	
			Limited demand	
[8]	Correlation; The coefficient of variation in demand volume; The proportion of periods with zero demand; Mean demand volume	–	–	–

As an unsupervised classification method, clustering can classify enormous data without any early knowledge about classes. For the time-series clustering, there are main three approaches, namely model-, shape-, and feature-based approaches [19]. In teleconsultation demand classification, discovering interesting patterns is the key point and guiding the service improvement is the final target. Therefore, the feature-based approaches will be applied in teleconsultation demand classification. Based on constructed features, hierarchal clustering makes a hierarchy of clusters using agglomerative or divisive algorithms [20]. In general, hierarchical algorithms are weak in terms of quality because clusters can’t be adjusted after splitting or merging a cluster. To overcome this weakness, the ensemble algorithm can be applied to obtain robust classification results upon hierarchal clustering methods. In the existing literature, the ensemble methods for clustering algorithms are often based on the proximity measures of individual clustering results [21, 22]. To enhance the proximity measures, individual clustering results can be one-hot encoded to remove the effect of numerical representation on the ensemble. One-hot encoding is more suitable for category result representation than numerical representation. After one-hot encoding, cosine similarity and K-means are used to identify the high similarity of individual clustering results for demand series. To obtained the ensemble clustering results, the high similarity of individual clustering results among demand series are presented in a network graph, in which the highly similar demand series are connected.

In a summary, the demand classification is proved to be effective to improve service in the previous studies. The existing classification methods may not suit teleconsultation demand classification. The clustering method is a flexible and widely applicable method for time series classification. Therefore, we would like to improve teleconsultation service by demand classification and classify teleconsultation demand by clustering methods. For robust classification results, an ensemble hierarchical method, combining hierarchal clustering, one-hot encoding, cosine similarity, K-means, and network graphs, is proposed.

## Methods

**Fig. 1:**
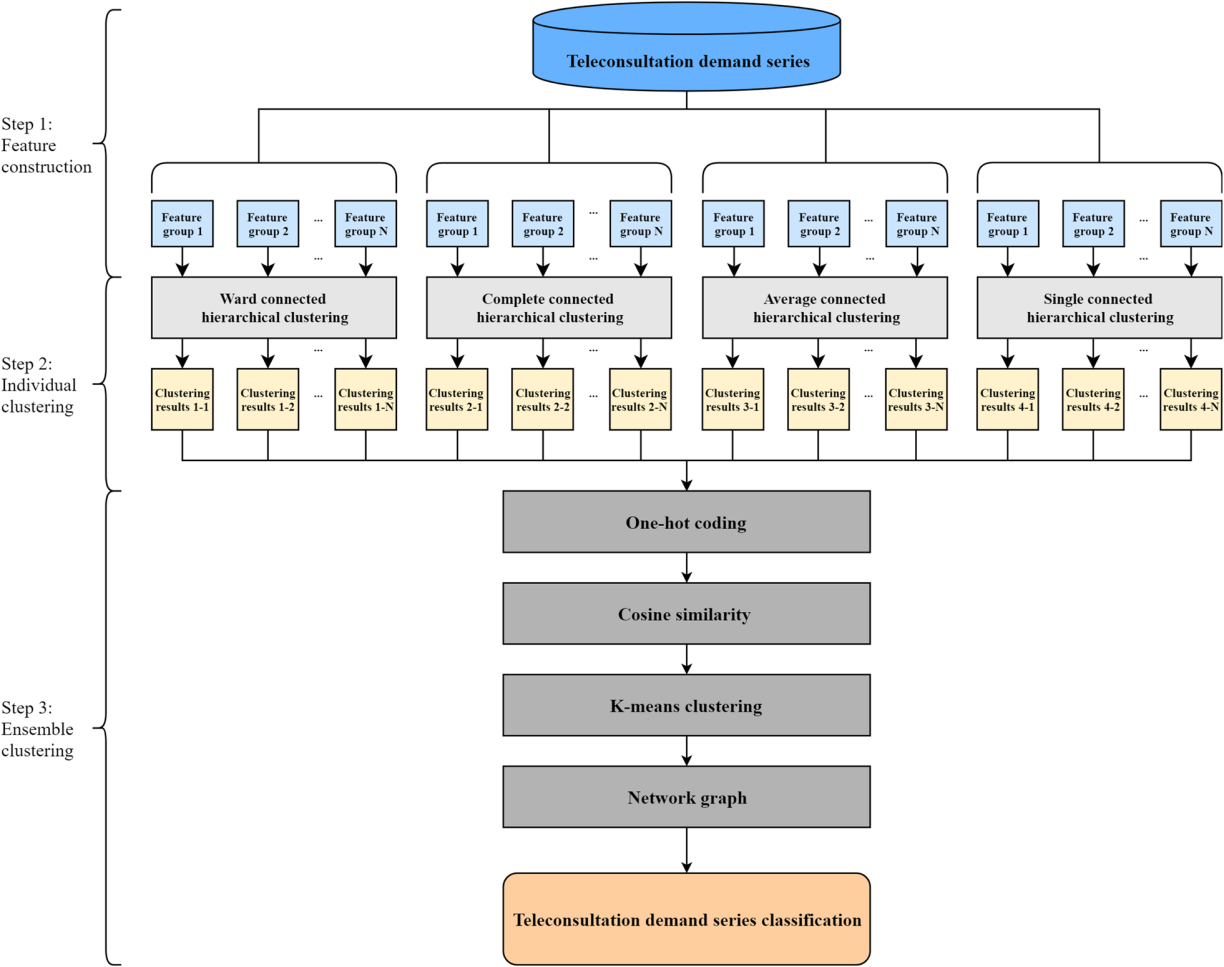
The framework of the proposed ensemble hierarchical clustering model

This section introduces the proposed ensemble hierarchical clustering method for teleconsultation demand classification. This method mainly combines the hierarchal clustering technique and ensemble algorithm. As shown in Fig. 1, there are three main steps in the proposed method, which are elaborated on below.

### Step 1: Feature construction

From the literature review, features for teleconsultation demand classification are constructed by corresponding definition and calculation. Each group of features is respectively inputted into the subsequent hierarchical clustering methods.

### Step 2: Individual clustering

To increase the diversity of the individual clustering results, four connected ways are respectively applied to build the hierarchical clustering methods. One group of features are inputted into a hierarchical clustering method to output individual clustering results.

### Step 3: Ensemble clustering

To obtain ensembled clustering results, the individual clustering results are first one-hot encoded, obtaining the high-dimensional sparse vectors. And then, the cosine similarity of those vectors is calculated to find the highly similar demand series in terms of individual clustering results. To divided the cosine similarity into three levels, high, middle, and low, the K-means clustering is applied. Based on the identified cosine similarity, the relationship of the corresponding demand series is presented in a network graph. In the network graph, nodes represent demand series and edges represent the cosine similarity of individual clustering results of the demand series. If the edges represent the high cosine similarity, the demand series, connected by edges in the network, can be categorized into one pattern.

## Data description and experimental design

### Dataset

The dataset in this study was provided by the National Telemedicine Center of China (NTCC). The dataset recorded the teleconsultation demands from January 1, 2018 to November 25, 2019. In the records, 65 departments provided teleconsultation services. Different departments have different demand sizes, as listed in Table 2. Overall, the demands of most departments are less than 500 during the 699-day observed period. The demands of 22 departments are less than 50. Because too little demand means highly sporadic demands, the data of those 22 departments are removed from the next experiments [10]. Thus, 43 series are applied in the next clustering experiments. Conveniently, these series are denoted as from Series-1 to Series-43 from the largest demand size to the smallest demand size. Series-1 represents the demand series of the respiratory department. The respiratory department has the most (4800) demands during the observed period.

**Table 2:** Teleconsultation demand size of medicine departments

Demand size	[1, 50]	[51, 500]	[501, 1000]	[1001, 2000]	[2001, 4000]	[4001, 5000]
Department quantity	22	21	12	5	3	2

### Experimental design

#### Feature selection

According to the literature review in Table 1, 58 features, belonging to six groups, were constructed, as listed in Additional file 1: Table A1.

#### Teleconsultation demand classification

The number of clusters is an important parameter in the clustering algorithm. To find a proper number of clusters, the results of previous studies are considered. For demand series classification, the four-category results in the previous study are proved to be effective [12, 13]. Therefore, consistent with the previous study, the number of clusters is set as 4 in hierarchical clustering methods. Besides, the number of clusters is set as 3 in the K-means clustering method, which is applied to divide the cosine similarity of individual clustering results into three levels, high, middle, and low.

To show the effectiveness of the proposed ensemble hierarchical clustering method for teleconsultation demand classification, existing clustering methods, including the traditional Syntetos’ and Boylan’s method, K-means, and hierarchical clustering methods, are applied as the benchmark methods. Furthermore, two traditional forecasting methods, the Croston and SBA methods, are performed. These two forecasting methods are proved to have different performances on different-pattern demands [13].

#### The criteria for teleconsultation service analysis

The teleconsultation services are analyzed from two perspectives, service efficiency and service quality. For the service efficiency, the demand days and service days are compared to show the level of scheduling teleconsultation doctors. One department doesn’t necessarily have teleconsultation demands every day. For one-day demands of one department demand, one doctor providing teleconsultation service is enough. Therefore, not lowering service quality, an efficient doctor scheduling means as few service days as possible. As for the service quality, mean patient waiting time is used to show the level of scheduling teleconsultation service. Mean patient waiting time is a traditional indicator for healthcare service quality [23, 24]. Not increasing service days, high-quality teleconsultation service means as a short mean patient waiting time as possible.

## Results and discussions

This section presents the empirical results and discussions. Specifically, the demand classification results are presented and discussed in “Teleconsultation demand classification” section, and the service analysis results are presented and discussed in “Teleconsultation service analysis” section.

### Teleconsultation demand classification

**Fig. 2:**
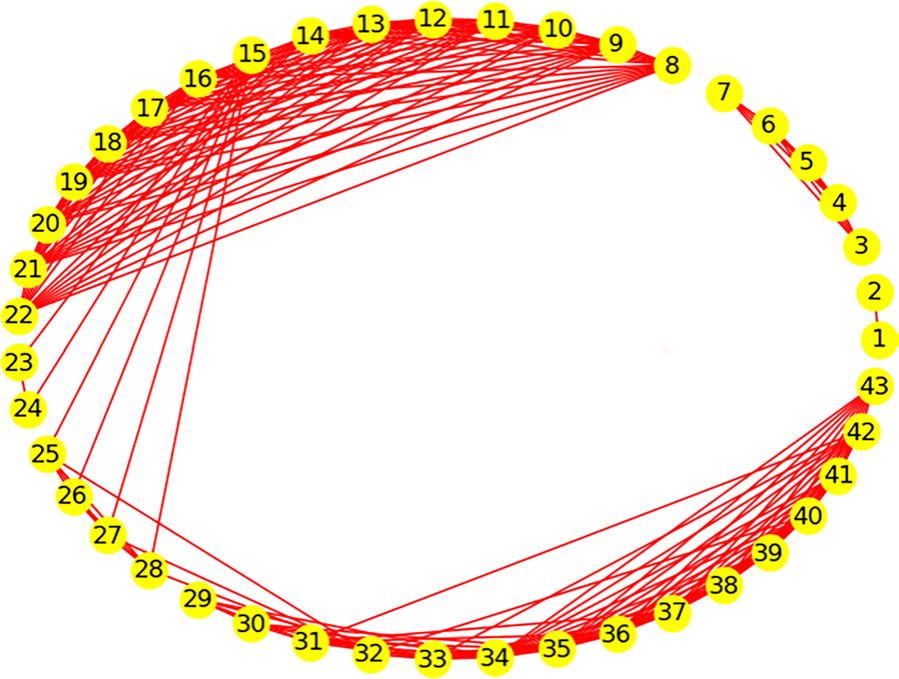
The network graph of demand series with the high cosine similarity of individual clustering results

To classify teleconsultation demand series, the proposed ensemble hierarchical clustering method is performed. In the proposed method, the divisions of the cosine similarity of individual clustering results are listed in Additional file 1: Table A2. The connections of demand series with the high cosine similarity of individual clustering results are shown in Fig. 2. Observing the edges, three groups of demand series can be found in Fig. 2, namely Series-1 and Series-2, Series-3 to Series-7, and Series-8 to Series-43. In these three groups, there is no edge between groups. Further to observe the group of Series-8 to Series-43, the edges are dense from Series-8 to Series-22 and from Series-25 to Series-43. The edges connecting the Series-23 and Series-24 with other series are sparse. Only demand series 15 has the high cosine similarity of individual clustering results with Series-23 and Series-24. Due to the high cosine similarity among Series-15 and Series-25 to Series-28, the two parts Series-8 to Series-22 and Series-25 to Series-43 are connected. Removing the rare edges of Series-15 with Series-23 to Series-28, there are three subgroups of demand series, namely series from 8 to 22, Series-23 and Series-24, and series from 25 to 43. In this case, the teleconsultation demand series are finally divided into five groups.

To further analyze the divisions of the demand series, the groups with only two series can be compared with the adjacent groups on the low cosine similarity of individual clustering results. Additional file 1: Fig. A1 presents the connection of demand series about the low cosine similarity of individual clustering results. From Additional file 1 Fig. A1(a), Series-23 and Series-24 have more connections of low cosine similarity with series from 25 to 43 than that with series from 8 to 22. Therefore, series 23 and 24 are more similar to the demand group of Series-8 to Series-22. Similarly, according to the number of low cosine similarity connections in Additional file 1: Fig. A1(b), series 1 and 2 are more similar to the group of Series-3 to Series-7.

**Fig. 3:**
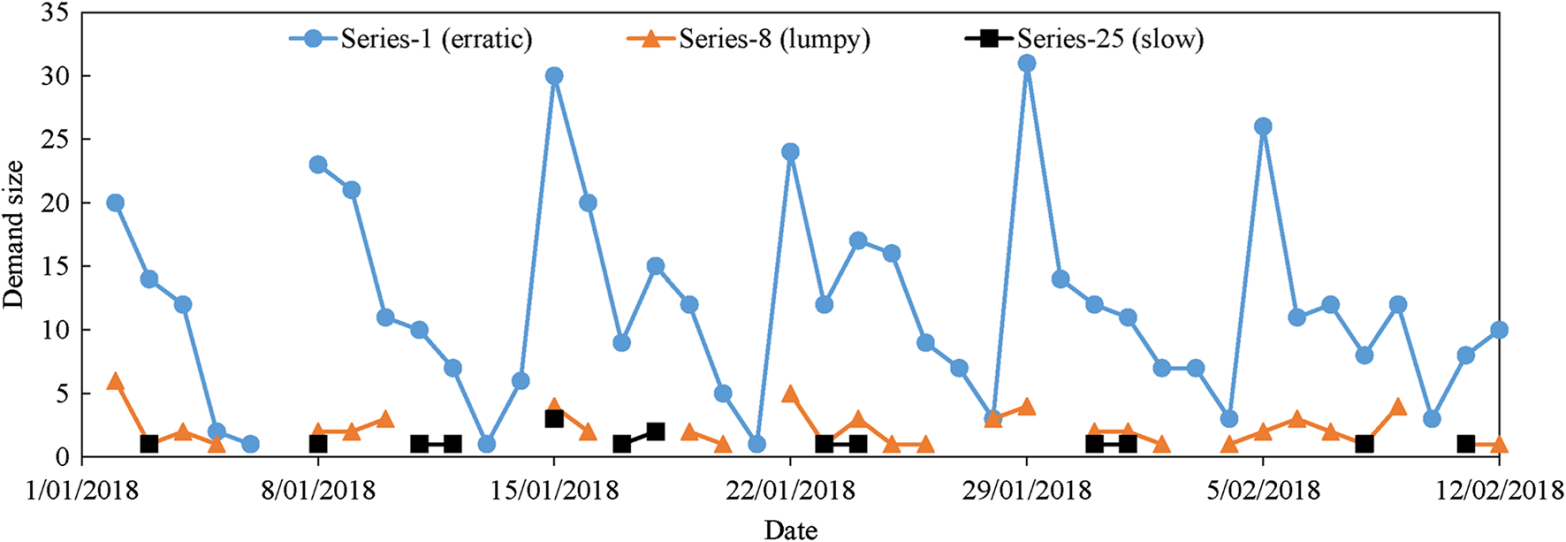
The demand changes of Series-1 (erratic), Series-8 (lumpy), and Series-25 (slow) with the date

According to the above categorization and similarity comparison and the definition of demand patterns, the five groups of teleconsultation demand include three patterns, as listed in Table 3. Series-1 to Series-7 are erratic demand (non-intermittent, and high volatility of demand size), Series-8 to Series-24 are lumpy demand (intermittent, and high volatility of demand size), and Series-25 to Series-43 is slow demand (intermittent, and low volatility of demand size) [10, 13]. Take Series-1, Series-8, and Series-25 for examples, Fig. 3 presents their demand change from 1/01/2018 to 12/02/2018. Series-1 conforms to the features of erratic demand, Series-8 conforms to the features of lumpy demand, and Series-25 conforms to the features of slow demand.

To prove the effectiveness of the proposed ensemble hierarchical clustering method, other existing clustering methods are performed, and the clustering results are compared in Additional file 1: Table A3. It can be found that the proposed method can divide teleconsultation into more groups. This can be explained by the ensemble of individual clustering results, which compares the demands more comprehensively. From series 1 to series 43, the demands show higher and higher intermittent and lower and lower size volatility. These changes of demand are gradual. More groups of demands can help to find the changes. Two criteria, the square coefficient of variation (CV^2^) of demand size and average inter-demand interval (AII), are applied to show the changes in demand patterns. These two criteria are the indicators in the traditional demand classification method [13].

**Table 3:** Classification results of teleconsultation demands

Demand	Erratic-1	Erratic-2	Lumpy-1	Lumpy-2	Slow
Series	1, 2	3–7	8–22	23, 24	25–43
CV^2^	0.40, 0.57	0.42–0.49	0.31–0.42, 0.65	0.37, 0.53	0.028–0.33
AII	1.59, 1.63	1.64–1.93	1.77–2.55, 3.76	2.87, 3.13	4.14–10.07

From the two criteria of demand patterns in Table 3, it can be observed that the threshold of CV^2^ lies in the range of 0.31–0.33 to identify the high level of demand size volatility for teleconsultation demand, and the threshold of AII lies in the range of 1.77–1.93 to identify the intermittent of the demand. For the lumpy-1 pattern, the unexpected CV^2^ (0.65) and AII (3.76) belong to the Series-15, which has unique connections with other demand series in Fig. 2. This is due to the series 15 representing the demand of the emergency department. The demands of the emergency department have higher uncertainty than the demand of other departments. Compared with lumpy-1, except for series 15, the AII of lumpy-2 are obviously higher, indicating the more intermittent of the demands of lumpy-2.

To prove the effectiveness of the classification results, two traditional intermittent demand forecasting methods, the Croston method, and the SBA method, are applied to forecast the department demands. These two methods have different forecasting accuracy on different category demands in SKU, and for demand with high size variability, SBA outperforms Croston [13]. Demand forecasting results of teleconsultation are listed in Additional file 1: Table A4. The demand forecasting accuracy of those two methods shows that SBA outperforms Croston on Series-1 to Series-10 but is inferior to Croston on Series-11 to Series-24, and the two methods are comparable on the remaining series. Those results can correspond to the results of the previous studies on different-pattern demand forecasting, demonstrating the effectiveness of the classification results.

### Teleconsultation service analysis

To find the defect of teleconsultation, the services are analyzed from service efficiency and service quality. The ratio of mean demand days to mean service days is used as the efficiency criteria, and mean patient waiting time is used as the quality criteria. Generally, larger ratios of efficiency are superior to the smaller ratios, and shorter mean patient waiting time is superior to the longer mean patient waiting time.

The calculation results of corresponding criteria of all demand and each pattern demand are listed in Table 4. The service efficiency ratio of all demand (none-classification) is lower than that of erratic demand and lumpy demand. Slow demand is highly intermittent, leading to the lowest efficiency ratio, near 1. For efficiency, the lumpy demands outperform other pattern demands. No matter whether the demands are classified or not, the mean patient waiting time is around 32 h, a day and a half. To further analyze the services, the mean intervals of inter-demand per week and mean length of the intervals are also calculated and the resulted are listed in Table 4. Overall, from all demand and each pattern demand, there is about an inter-demand interval in a week. The mean interval lengths are in the range of 1.74–6.72 days.

**Table 4:** The teleconsultation service analysis

Criteria (actual)	Demand			
	All	Erratic	Lumpy	Slow
Mean demand days (MDD)	275.40	533.86	349.65	113.74
Mean service days (MSD)	195.79	377.43	231.71	96.74
Ratio = MDD/MSD	1.41	1.41	1.51	1.18
Mean waiting time (h)	31.78	30.86	32.82	33.33
Mean intervals per week	1.17	0.99	1.52	0.96
Mean internal length (day)	4.18	1.74	2.37	6.72

From Table 4, the defect of teleconsultation is unobvious. For further comparison, the fixed strategies of service arrangement are introduced to analyze teleconsultation service, and the results are listed in Table 5. A fixed strategy, based on the mean service days in Table 4, means the frequency of service is fixed in a week. Ideal service times are equal to the service times per week multiplied by the weeks. Under the fixed strategy, the service day is determined according to the weekly demand distribution of teleconsultation in Additional file 1: Fig. A2. Generally, a teleconsultation service is provided on the day with more demands to lower the waiting time of patients. To simplify the calculation of the mean waiting time under the fixed strategies, 5:00 p.m. is set as the service time. The real daily demands and service time are presented in Additional file 1: Figs. A3 and A4.

**Table 5:** The teleconsultation service analysis under the fixed strategies

Service	Demand				
	All	Erratic	Lumpy	Lumpy	Slow
Fixed strategy (FS)	2 Services/week	4 Services/week	3 Services/week	2 Services/week	1 Service/ week
Ideal service times	190	380	285	190	95
Service day	Wednesdays and Fridays	Mondays to Thursdays, and Saturdays	Mondays, Wednesdays, and Fridays	Tuesdays, Fridays	The next day of the demand day
Mean waiting time (h) under the FS	33.14	9.15	17.41	29.46	29.44

(1) Ideal service times = (Service times/week) * (weeks). During the observed period, there are 99 weeks and four 7-day holidays (two Spring Festival and two National Day). Because teleconsultation demand on holidays almost decreases to zero, the number of weeks providing service is 95. (2) Waiting time depends on the demand arriving time and the service providing time. Considering the time distributions of teleconsultation demand arriving and service providing in Additional file 1: Figs. A2 and A3, 5:00 p.m. is set as the service time to calculate the waiting time. On Saturdays, the service time is noon

From Table 4 and Table 5, the service analysis before and after demand classification under the fixed service strategies have different conclusions. Without demand classification, the mean service days of teleconsultation is 196, close to the ideal 190 days. The mean patient waiting time is 31.78 h, close to that of under the fixed strategies 33.14 h. These comparisons indicate that the efficiency and quality of teleconsultation services needn’t be improved. However, after demand classification, the conclusion is different from that of before demand classification.

For erratic demand, although the actual mean service days (377.43) are comparable to ideal service days, the actual mean patient waiting time is 21.71 h longer (almost 3.4 times) than the ideal mean waiting time. Therefore, the focus of service improvement of erratic demand is to decrease the mean waiting time of patients. For lumpy demand, the actual service days (231.71) are less than ideal service days (285) applying 3 services/week strategy, and more than ideal service days (190) applying 2 services/week strategy. The actual mean patient waiting time of lumpy demand is respectively 15.41 h and 3.36 h longer than that of applying 3 services/week strategy and 2 services/week strategy. Therefore, the service improvement for lumpy demand is to decrease both the service days and mean patient waiting time. For slow demand, both the actual mean service days and mean patient waiting time are comparable to these under the fixed strategy. But if the service time is fixed on the day when the demand occurs, the ideal mean waiting can be further decreased.

From the above analysis, the different results of service analysis before and after demand classification demonstrate the importance of demand classification for teleconsultation management and suggest the effectiveness of the proposed ensemble hierarchical clustering methods for teleconsultation demand classification.

## Conclusions

To improve teleconsultation management, the demands are classified by the proposed ensemble hierarchical clustering method, and the service efficiency and quality are analyzed before and after demand classification. For the effective demand classification, the proposed method is a method- and feature-based ensemble clustering. The ensemble involves one-hot encoding, cosine similarity, and network graph representation. Utilizing real teleconsultation data as sample data and general clustering methods as benchmark models, empirical results show the effectiveness of the proposed ensemble hierarchical clustering method.

By the proposed method, teleconsultation demands are categorized into five groups, in three patterns, erratic, lumpy, and slow. The significance of the demand classification is proved by the comparison between the service analysis without demand classification and the service analysis after demand classification. Results show that service analysis after demand classification can reveal the problems on teleconsultation service of each pattern demand, and provide suggestions for the service improvement.

Despite the suggestions for the service improvements proposed in this study, there is still room for further research. For example, the mean waiting time under fixed strategies is calculated under the fixed service time. This may not be the reality. To further improve the service, different scheduling strategies can be studied in teleconsultation scheduling.

## Supplementary Information

**Additional file 1.Table A1**. Selected features for teleconsultation demand classification.** Table A2**. The divisions of the cosine similarity of individual clustering results.** Table A3**. The clustering results of different methods.** Figure A1**. The network graph of demand series with the low cosine similarity of individual clustering results.** Table A4**. Demand forecasting results of Croston method and SBA method. **Figure A2**. Weekly demand of teleconsultation.** Figure A3**. Daily demands of teleconsultation.** Figure A4**. Distribution of teleconsultation service providing time in a day.

## Data Availability

The data underlying this article were provided by the National Telemedicine Center of China under license. Data will be shared on request to the corresponding author with the permission of the National Telemedicine Center of China.

## Abbreviations

AII: Average inter-demand interval
CV^2^: The square coefficient of variation
SKU: Stock keeping unit
NTCC: National Telemedicine Center of China
