# Supplementary material for: Teleconsultation demand classification and service analysis
Source: BMC Med Inform Decis Mak. 2021 Aug 21;21:245. doi: 10.1186/s12911-021-01610-x (PMC8379888; doi:10.1186/s12911-021-01610-x)
Supplement: Supplementary file 1 — Additional file 1.Table A1. Selected features for teleconsultation demand classification. Table A2. The divisions of the cosine similarity of individual clustering results. Table A3. The clustering results of different methods. Figure A1. The network graph of demand series with the low cosine similarity of individual clustering results. Table A4. Demand forecasting results of Croston method and SBA method. Figure A2. Weekly demand of teleconsultation. Figure A3. Daily demands of teleconsultation. Figure A4. Distribution of teleconsultation service providing time in a day. [file 12911_2021_1610_MOESM1_ESM.docx]

Table A1 Selected features for teleconsultation demand classification.

| Category  (features) | Feature |  | Category  (features) | Feature |
| --- | --- | --- | --- | --- |
| Demand volume (10) | Total |  | The number of zero demand periods in the last 8 periods (8) | Max |
|  | Max |  |  | 75th percentile |
|  | 75th percentile |  |  | Median |
|  | Median |  |  | 25th percentile |
|  | 25th percentile |  |  | Min |
|  | Min |  |  | Mode |
|  | Mode |  |  | Mean |
|  | Mean |  |  | Standard deviation |
|  | Standard deviation |  | The number of zero demand periods in the last 13 periods (8) | Max |
|  | CV^2^ |  |  | 75th percentile |
| Inter-demand interval size (3) | Number of zero-demand days |  |  | Median |
|  | Number of intervals |  |  | 25th percentile |
|  | Average interval |  |  | Min, Mode |
| Non-zero demand periods (9) | Number of lengths |  |  | Mean |
|  | Max |  |  | Standard deviation |
|  | 75th percentile |  | Correlation (20) | Lag step=1，AC=PAC, Prob |
|  | Median |  |  | Lag step=2，AC, PAC, Prob |
|  | 25th percentile |  |  | Lag step=3，AC, PAC, Prob |
|  | Min |  |  | Lag step=4，AC, PAC, Prob |
|  | Mode |  |  | Lag step=5，AC, PAC, Prob |
|  | Mean |  |  | Lag step=6，AC, PAC, Prob |
|  | Standard deviation |  |  | Lag step=7，AC, PAC, Prob |

Table A2 The divisions of the cosine similarity of individual clustering results

| The cosine similarity | Values |
| --- | --- |
| High | 0.00, 0.04, 0.08, 0.13, 0.17, 0.21, 0.25, 0.29, 0.33, 0.38 |
| Middle | 0.42, 0.46, 0.50, 0.54, 0.58, 0.63, 0.67, 0.71 |
| Low | 0.75, 0.79, 0.83, 0.88, 0.92, 0.96, 1.00 |

Table A3 The clustering results of different methods

| Method | Syntetos | Boylan | K-means | Hierarchical clustering | | | | Proposed |
| --- | --- | --- | --- | --- | --- | --- | --- | --- |
|  |  |  |  | Ward | Complete | Average | Single |  |
| Classification results | Lumpy:Series 1, 5, 14, 15, 24 | non-intermittent: series 1-4 | series 1-2 | series 1-2 | series 1-2 | series 1-2 | series 1 | series 1-2 |
|  |  |  | series 3-7 | series 3-7 | series 3-5 | series 3 | series 2 | series 3-7 |
|  | Slow: Series 2-4, 6-13, 16-23, 25-43 | Intermittent :series 5-43 | series 8-22 | series 8-24 | series 6-10 | series 4-7 | series 3 | series 8-22 |
|  |  |  | series 23-43 | series 25-43 | series 11-43 | series 8-43 | series 4-43 | series 22-23 |
|  |  |  |  |  |  |  |  | series 25-43 |


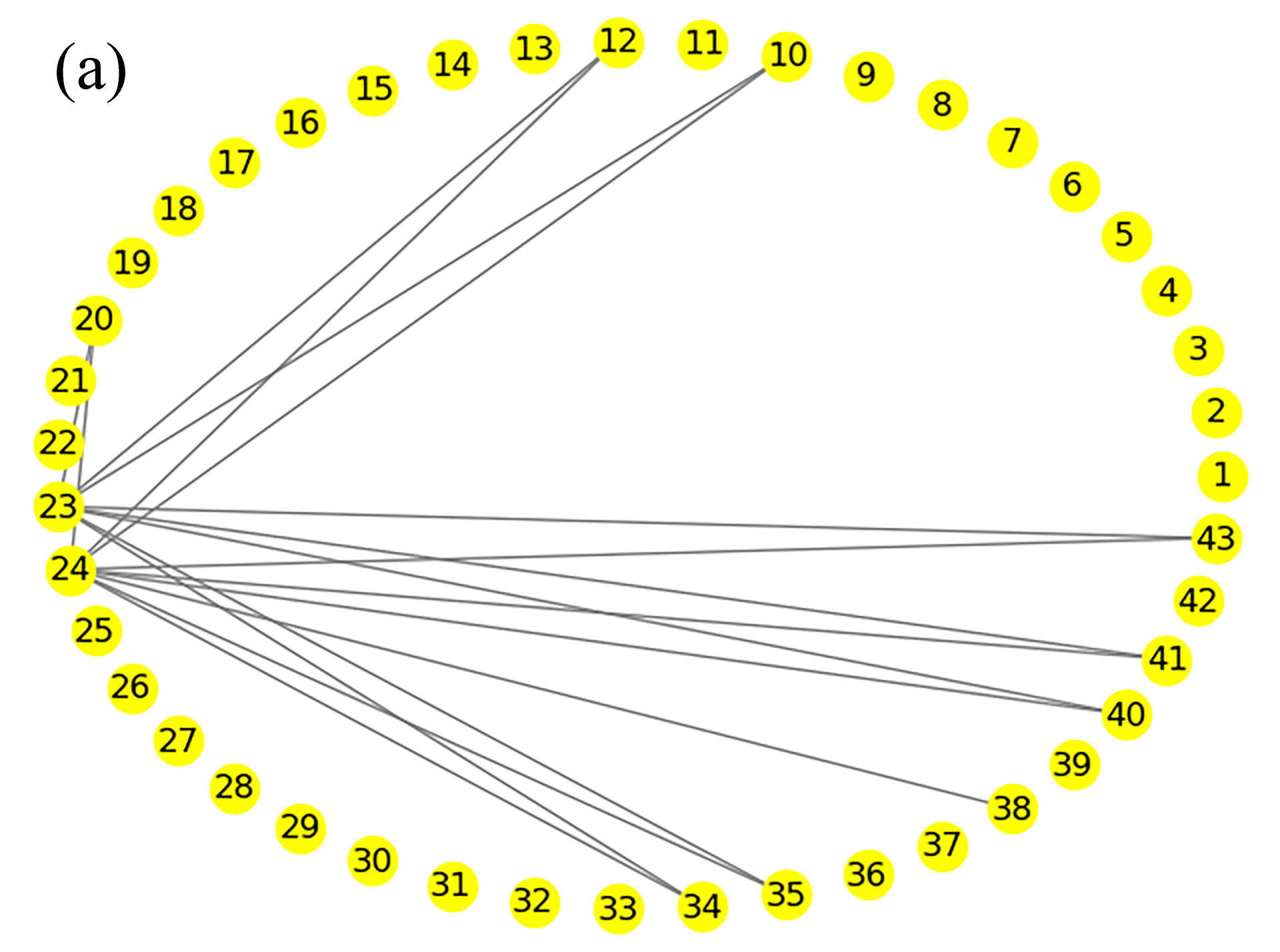


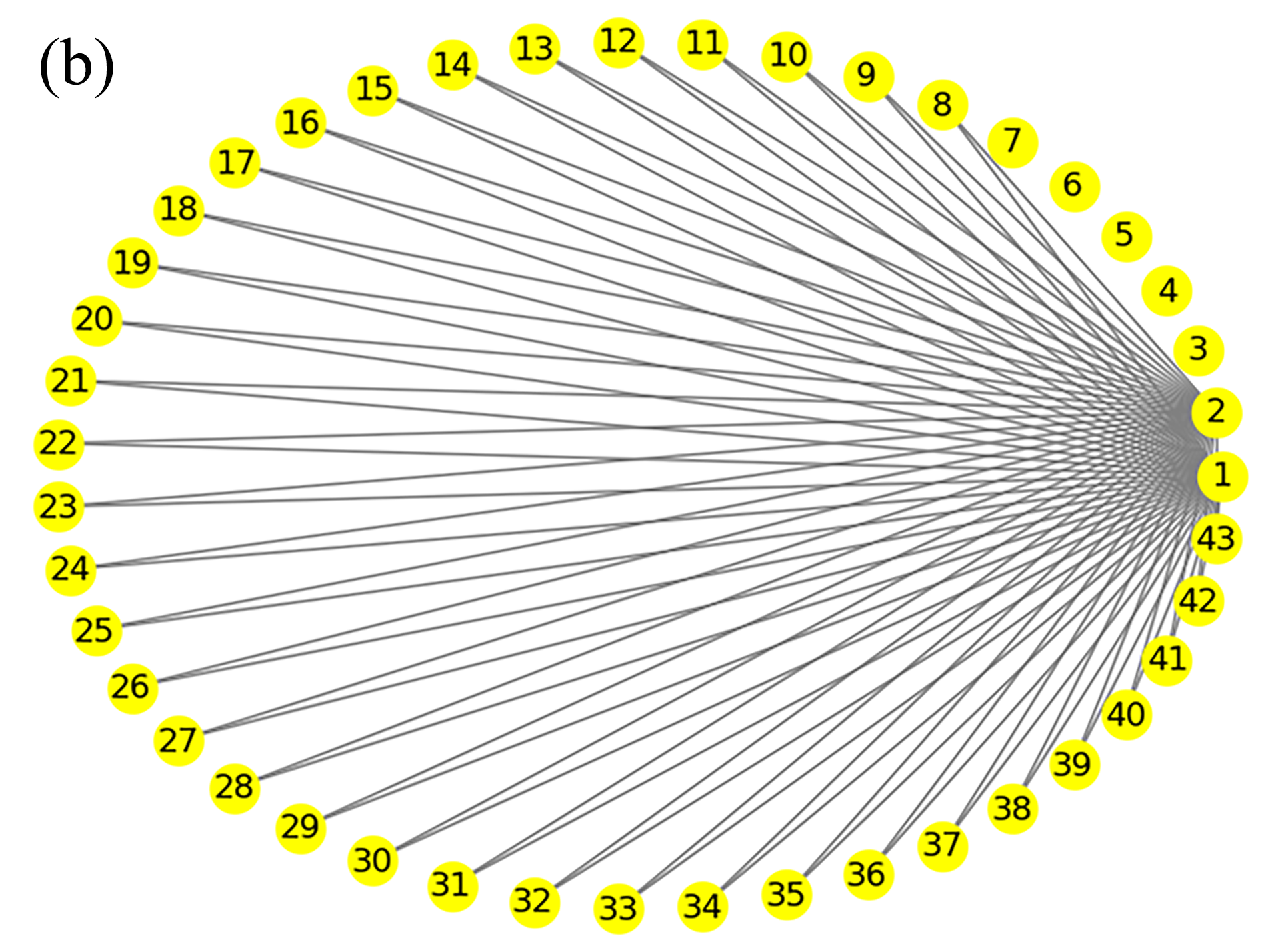


Figure A1 The network graph of demand series with the low cosine similarity of individual clustering results.

Table A4 Demand forecasting results of Croston method and SBA method.

| Series | Croston model | |  | SBA model | | MSE_C_ - MSE_S_ | MAE_C_ - MAE_S_ |
| --- | --- | --- | --- | --- | --- | --- | --- |
|  | MSE | MAE |  | MSE | MAE |  |  |
| 1 | 35.35 | 4.39 |  | 27.91 | 3.86 | 7.44 | 0.53 |
| 2 | 21.20 | 3.70 |  | 18.44 | 3.51 | 2.76 | 0.19 |
| 3 | 20.22 | 3.47 |  | 16.25 | 3.01 | 3.97 | 0.46 |
| 4 | 11.20 | 2.69 |  | 9.15 | 2.32 | 2.06 | 0.38 |
| 5 | 10.96 | 2.34 |  | 8.84 | 2.12 | 2.12 | 0.22 |
| 6 | 8.64 | 2.27 |  | 5.98 | 1.95 | 2.66 | 0.32 |
| 7 | 7.11 | 2.15 |  | 5.99 | 1.90 | 1.13 | 0.25 |
| 8 | 3.10 | 1.42 |  | 2.66 | 1.25 | 0.45 | 0.17 |
| 9 | 6.24 | 1.97 |  | 5.72 | 1.71 | 0.52 | 0.26 |
| 10 | 3.09 | 1.43 |  | 2.53 | 1.22 | 0.56 | 0.21 |
| 11 | 1.77 | 1.05 |  | 1.95 | 1.07 | -0.18 | -0.01 |
| 12 | 2.47 | 1.14 |  | 2.63 | 1.27 | -0.16 | -0.13 |
| 13 | 1.98 | 1.18 |  | 2.33 | 1.18 | -0.35 | 0.00 |
| 14 | 2.18 | 1.07 |  | 2.47 | 1.08 | -0.30 | -0.01 |
| 15 | 2.91 | 1.16 |  | 2.93 | 1.14 | -0.02 | 0.02 |
| 16 | 1.62 | 0.96 |  | 1.71 | 0.94 | -0.09 | 0.03 |
| 17 | 1.47 | 0.95 |  | 1.42 | 0.88 | 0.05 | 0.07 |
| 18 | 2.30 | 1.18 |  | 2.39 | 1.06 | -0.09 | 0.11 |
| 19 | 1.96 | 1.14 |  | 2.09 | 1.11 | -0.13 | 0.02 |
| 20 | 1.92 | 1.04 |  | 2.05 | 1.04 | -0.13 | 0.00 |
| 21 | 1.12 | 0.80 |  | 1.27 | 0.80 | -0.14 | -0.01 |
| 22 | 0.91 | 0.78 |  | 0.91 | 0.77 | 0.00 | 0.02 |
| 23 | 0.72 | 0.65 |  | 0.75 | 0.61 | -0.03 | 0.04 |
| 24 | 1.14 | 0.75 |  | 1.19 | 0.69 | -0.05 | 0.07 |
| 25 | 0.47 | 0.53 |  | 0.47 | 0.53 | 0.00 | 0.00 |
| 26 | 0.43 | 0.52 |  | 0.43 | 0.51 | 0.00 | 0.01 |
| 27 | 0.28 | 0.35 |  | 0.28 | 0.35 | 0.00 | 0.01 |
| 28 | 0.26 | 0.42 |  | 0.25 | 0.41 | 0.00 | 0.01 |
| 29 | 0.17 | 0.32 |  | 0.16 | 0.30 | 0.01 | 0.02 |
| 30 | 0.53 | 0.54 |  | 0.53 | 0.52 | 0.00 | 0.02 |
| 31 | 0.35 | 0.42 |  | 0.35 | 0.40 | 0.00 | 0.02 |
| 32 | 0.39 | 0.40 |  | 0.39 | 0.39 | 0.00 | 0.01 |
| 33 | 0.54 | 0.46 |  | 0.55 | 0.46 | 0.00 | 0.00 |
| 34 | 0.33 | 0.37 |  | 0.33 | 0.36 | 0.00 | 0.00 |

MSE_C_ : the MSE of the Croston model; MSE_S_ :the MSE of the SBA model; MAE_C_ : the MAE of the Croston model; MAE_S_ : the MAE of the SBA model.


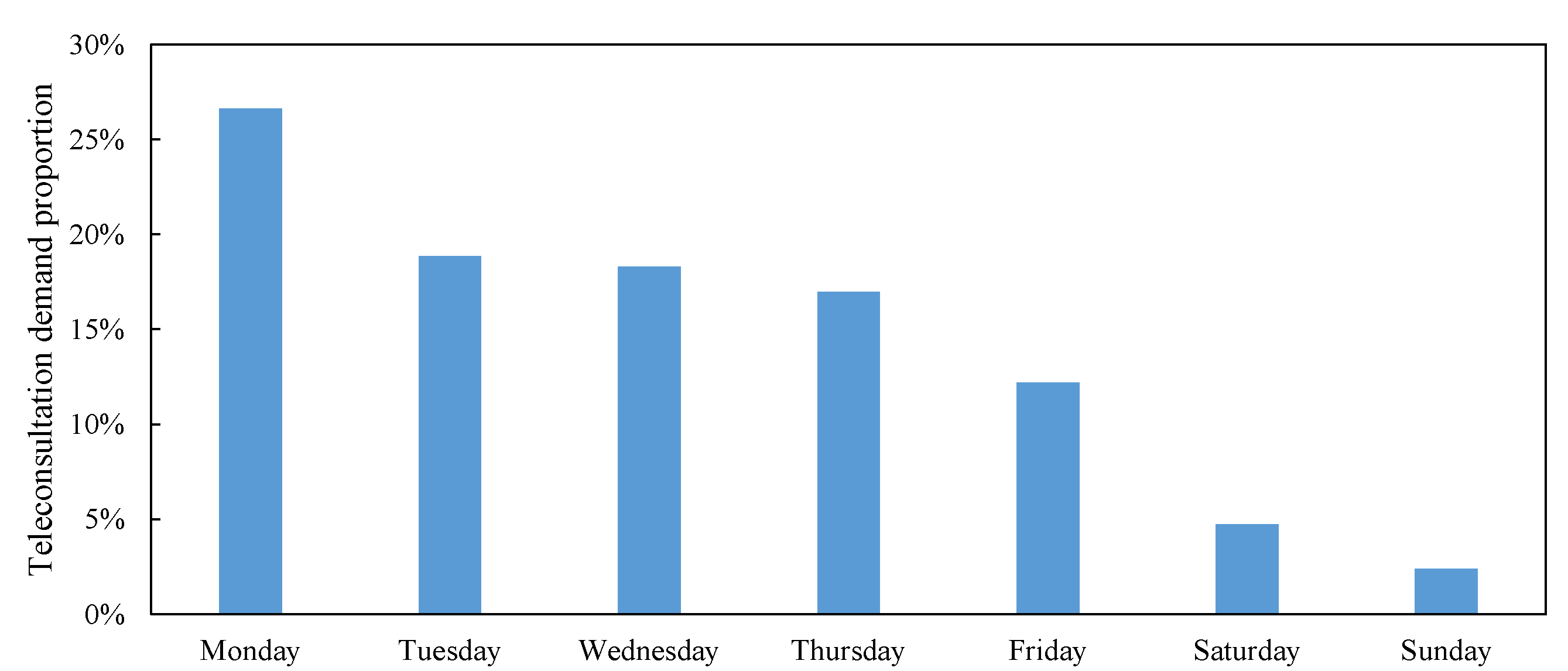


Figure A2 Weekly demand of teleconsultation.


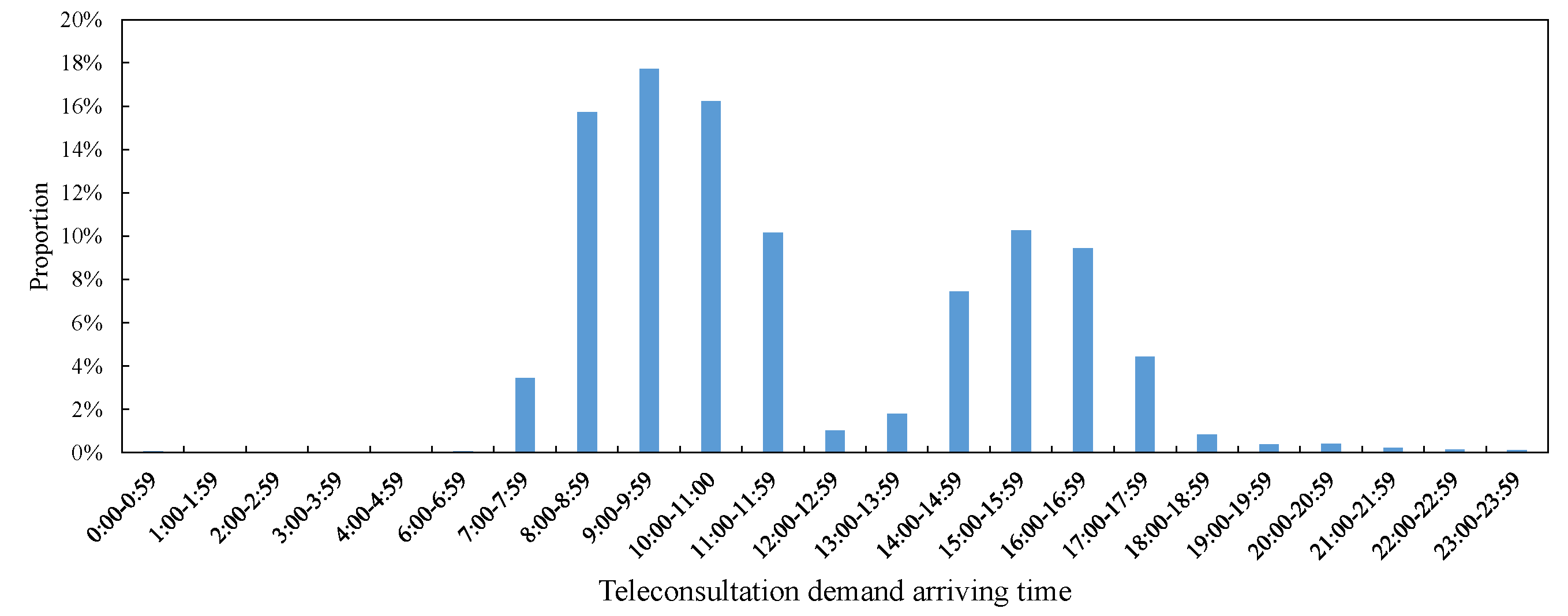


Figure A3 Daily demands of teleconsultation.


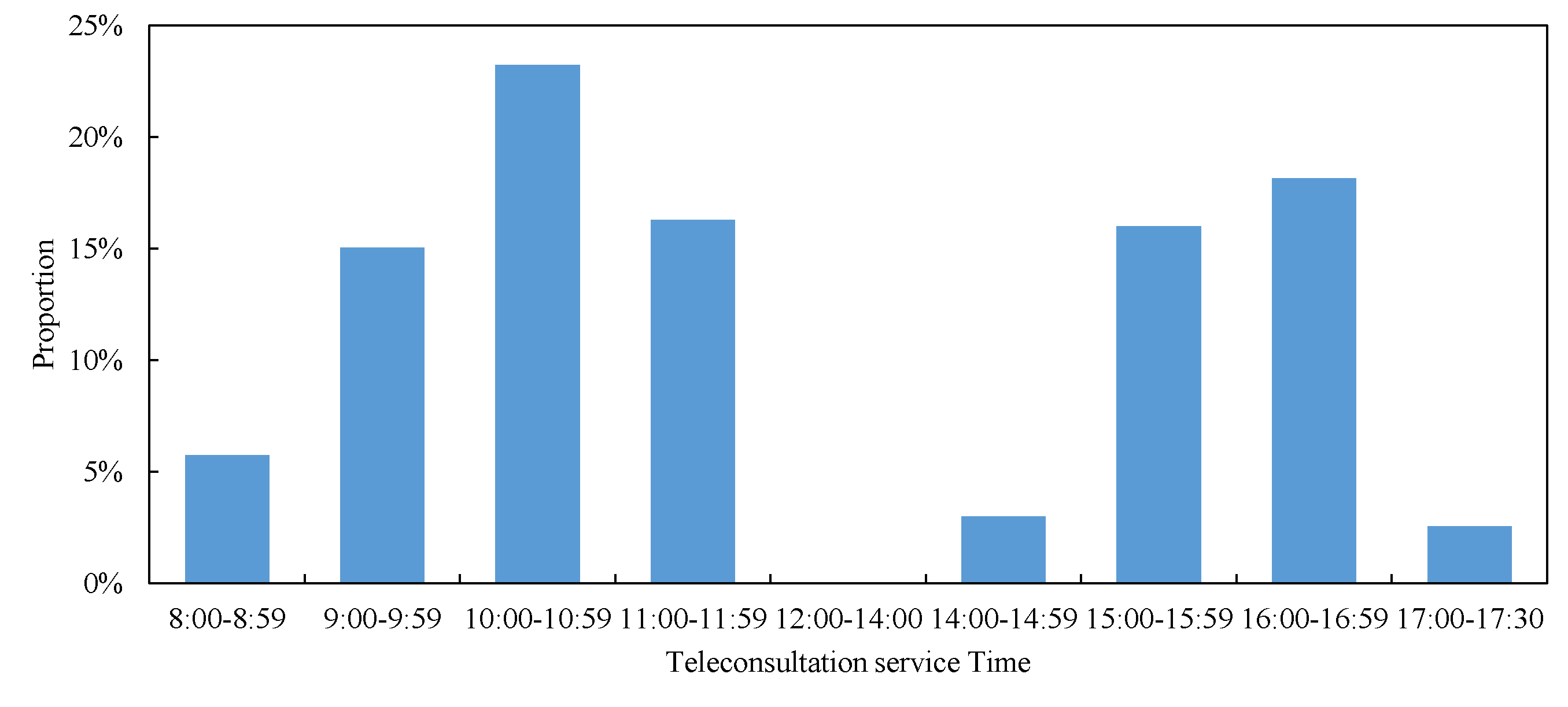


Figure A4 Distribution of teleconsultation service providing time in a day.
